# Outdoor biting and pyrethroid resistance as potential drivers of persistent malaria transmission in Zanzibar

**DOI:** 10.1186/s12936-022-04200-y

**Published:** 2022-06-07

**Authors:** Revocatus M. Musiba, Brian B. Tarimo, April Monroe, Dickson Msaky, Halfan Ngowo, Kimberly Mihayo, Alex Limwagu, Godlove T. Chilla, Gasper K. Shubis, Ahmada Ibrahim, George Greer, Juma H. Mcha, Khamis A. Haji, Faiza B. Abbas, Abdullah Ali, Fredros O. Okumu, Samson S. Kiware

**Affiliations:** 1 Ifakara Health Institute, Dar es Salaam, Tanzania; 2 PMI VectorWorks Project, Johns Hopkins Center for Communication Programs, Baltimore, MD USA; 3 US President’s Malaria Initiative, U.S. Agency for International Development, Dar es Salaam, Tanzania; 4 Zanzibar Malaria Elimination Programme, Zanzibar, Tanzania; 5 Pan African Mosquito Control Association (PAMCA), Nairobi, Kenya

**Keywords:** Entomology, Malaria transmission, Novel tools, Pyrethroids, Insecticide resistance

## Abstract

**Background:**

Low-level of malaria transmission persist in Zanzibar despite high coverage of core vector control interventions. This study was carried out in hot-spot sites to better understand entomological factors that may contribute to residual malaria transmission in Zanzibar.

**Methods:**

A total of 135 households were randomly selected from six sites and consented to participate with 20–25 households per site. Mosquito vector surveillance was carried out indoors and outdoors from 6:00 pm–7:00 am using miniaturized double net trap (DN-Mini™). Additional collections were done indoors using mouth aspirators to retrieve resting mosquitoes from wall and ceiling surfaces, and outdoors using resting bucket and pit traps. All collected mosquitoes were morphologically and genetically (PCR) analysed in the laboratory. All collected anopheline and blood-fed mosquitoes were analysed for sporozoite infection and blood meal host preferences by Circumsporozoite Protein ELISA and blood meal ELISA, respectively. The differences between indoor and outdoor mosquito biting rates were analysed using generalized linear mixed models. Levels of resistance to commonly used insecticides were quantified by WHO susceptibility tests.

**Results:**

Out of 704 malaria vectors collected across 135 households, PCR analysis shows that 98.60% were *Anopheles arabiensis*, 0.6% *Anopheles merus* and 0.6% *Anopheles gambiae *sensu stricto*.* Sporozoite ELISA analysis indicates that all mosquitoes were negative for the malaria parasite. The results show that more *An. arabiensis* were collected outdoor (~ 85%) compared to indoor (~ 15%). Furthermore, large numbers of *An. arabiensis* were caught in outdoor resting sites, where the pit trap (67.2%) collected more mosquitoes compared to the outdoor DN-Mini trap (32.8%). Nearly two-thirds (60.7%) of blood-fed mosquitoes had obtained blood meals from non-human hosts. Mosquitoes displayed non-uniform susceptibility status and resistance intensity among the tested insecticides across the study sites to all WHO recommended insecticides across the study sites.

**Conclusion:**

This study suggests that in contexts such as Zanzibar, testing of novel techniques to complement indoor protection and targeting outdoor biting and/or resting mosquitoes, may be warranted to complement existing interventions and contribute to malaria elimination efforts. The study highlights the need to implement novel interventions and/or adaptations of strategies that can target outdoors biting mosquitoes.

## Background

Current vector control interventions, notably long-lasting insecticidal nets (LLINs) and indoor residual spraying (IRS), have contributed to significant reductions in malaria burden [1–4]. The archipelago of Zanzibar which constitutes Unguja and Pemba Islands and part of the United Republic of Tanzania, located on the coast of East Africa, has experienced a considerable reduction in malaria burden [4–6]. Reduction in parasite prevalence has been observed from historical levels of 75% to < 1.5% by 2009 [5], with a further reduction to < 0.1% by 2016 [6]. This decline in malaria cases in Unguja and Pemba Islands is attributed to wide and sustained use of malaria control measures, such as indoor residual spraying (IRS) of households, LLINs, the use of rapid diagnostic tests (RDTs), and case management with artemisinin-based combination therapy (ACT) [7]. With such low parasite prevalence rates, Zanzibar can feasibly aim for elimination of the disease [8].

LLINs and IRS effectively reduce malaria transmission by targeting indoor feeding (endophagic) and resting (endophilic) mosquitoes [9, 10]. These interventions offer robust individual protection from indoor malaria vectors and can confer community protection when high levels of coverage are achieved [11]. The sustained and wide range use of LLINs and IRS has led to changes in the vector population in some contexts due to the development of physiological or behavioural resistance [7]. Physiological resistance involves the capability of the vector to physiologically tolerate the insecticide present in LLINs and IRS leading to a prolonged life span. While behavioural resistance involves behavioural plasticity from endophilic, endophagic, and anthropophilic mosquitoes to more exophilic, exophagic, and zoophilic mosquitoes to escape from contact with insecticides in LLINs and IRS [7]. In both instances, the effectiveness of vector control interventions may be diminished.

Data collected in 2013 and 2014 indicated that the predominant malaria vector species were *Anopheles arabiensis* (76.0%) in Pemba and *Anopheles gambiae* (83.5%) in Unguja (data collected between June 2013 and February 2014 from three sites in Pemba island and four sites in Unguja) [12]. The FY 2015 President Malaria initiate (PMI) Malaria Operational Plan, based on Zanzibar Malaria Elimination Programme (ZAMEP) mosquito collections, reported similar findings. However, recent data from routine entomological surveillance conducted in Unguja by ZAMEP show an increase in the numbers of *An. arabiensis* collected relative to *An. gambiae *sensu stricto (s.s.). Furthermore, increasing outdoor biting exposure has been observed in both Pemba and Unguja evidenced from recent human-landing catches done indoors and outdoors (ZAMEP report, unpublished data). This outdoor biting behaviour has been shown to occur early in the evening, peaking as early as 8–10 pm (unpublished data from ZAMEP). In addition to endophagic and exophagic behaviour, *Anopheles* mosquitoes in Zanzibar have been shown to be resistant to pyrethroid-based insecticides [13, 14]. In 2012, there was a policy change in the insecticide used in IRS from pyrethroids class to carbamate class specifically bendiocarbs, which was later abandoned in 2014 due to its short residual life span that led to increased IRS operational costs. Currently, pirimiphos-methyl is the insecticide of choice for IRS in Zanzibar and there has not been any reported resistance against it. Increasing insecticide resistance is a major hurdle for malaria control efforts in sub-Sahara Africa (SSA). Therefore, routine surveillance is essential to ensure early detection of resistance against insecticide(s) used and forms the basis on the choice of insecticide to be used for LLINs and IRS.

Recent data from Zanzibar show that malaria cases have been mainly focalized in specific areas with high vector abundance and possibly, where people have recently travelled from mainland Tanzania [8]. To better understand potential entomological drivers of persistent low-level malaria transmission in Zanzibar, investigation on vector biting and resting patterns, host preferences, and levels of resistance to commonly used insecticides was carried out. This work was carried out as part of a larger research project investigating both entomological and human behavioural factors that may be contributing to residual malaria transmission in Zanzibar. The results on human behaviour and human-vector interaction are presented in accompanying studies [15]. To complement ongoing routine mosquito surveillance carried by ZAMEP, this study was carried out in sites not currently covered by ZAMEP, focusing on hot spots, and utilized higher resolution sampling approach using a different data collection method. The data collection activities were also managed by an electronic-based entomological system making it easier to link these datasets with other sources of data from the same area at the household level.

## Methods

### Study sites

**Fig. 1:**
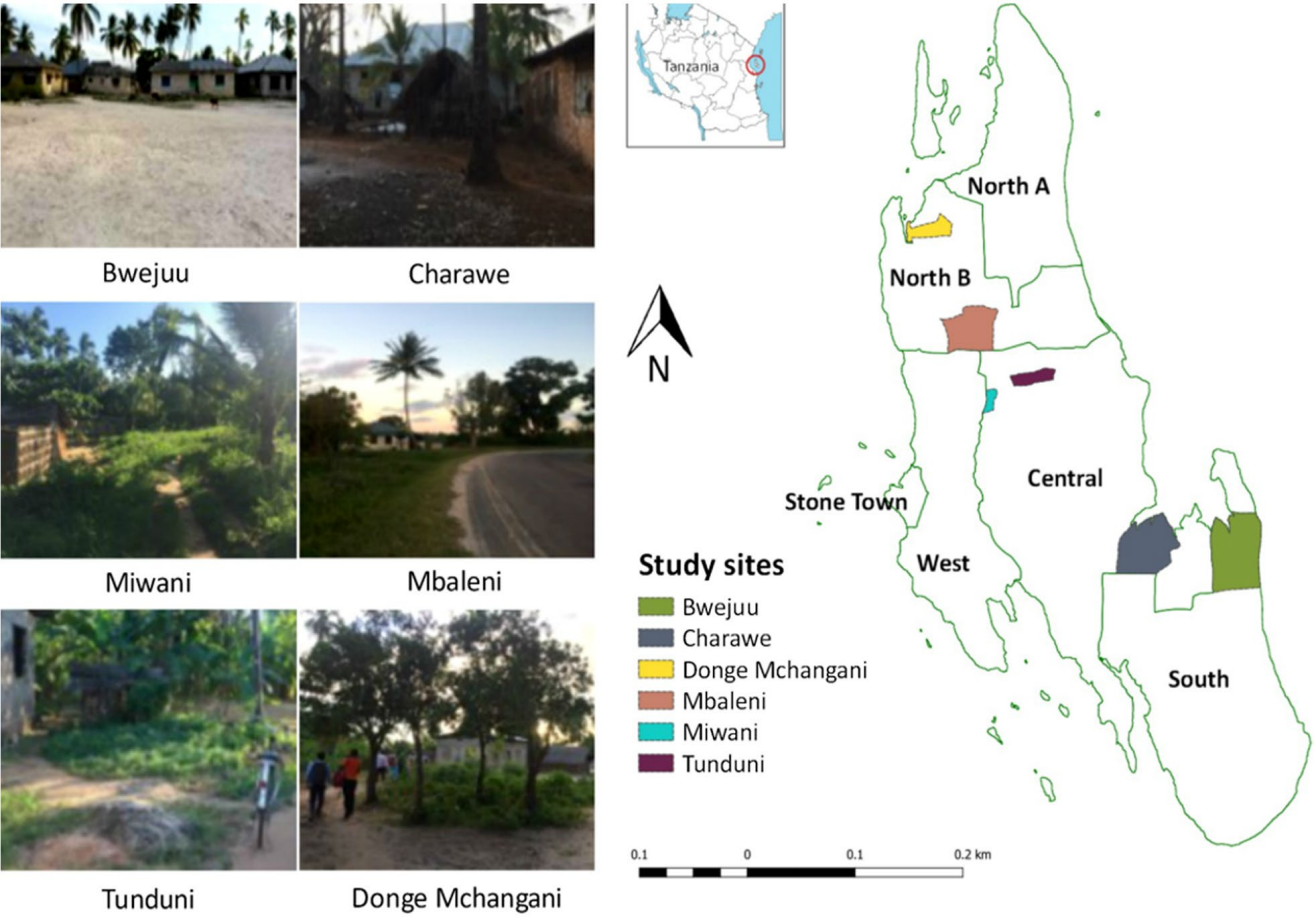
The figure presents the six study sites in Unguja and a snapshot of their characteristics

This study was conducted in Unguja (6.1357° S, 39.3621° E), the main island in the archipelago of Zanzibar. Zanzibar is currently in the pre-elimination stage and field evidence suggest that remaining cases are focalized, coinciding with high vector abundance [8], and areas where residents frequently travel to mainland Tanzania. Six shehias across four districts were selected based on high annual parasite incidence level (i.e., 5/1000 number of new infections per year per 1000 population) and having received IRS in the past year (2016). In Zanzibar, *shehia* is defined as “the local government authority area and the lowest government administrative structure at the community level’’. These six-selected shehias were Miwani, Mbaleni, Bwejuu, Donge-Mchangani, Tunduni, and Charawe as shown in Fig. 1. Furthermore, Zanzibar Malaria Elimination Programme (ZAMEP) was currently not conducting routine mosquito surveillance in the selected sites.

### Study design and sample size

Entomological information was collected to assess vector biting and resting patterns, host preferences, and levels of resistance to commonly used insecticides. A total of 135 households across the six shehias consented to participate in mosquito collection. Mosquito collections were carried out indoors and outdoors from 6:00 pm–7:00 am, using standardized exposure-free volunteer-baited trapping methods (primary outcome measure) [16, 17]. Each household was visited eight times over a period of 12 months, from December 2016–December 2017. This sampling plan was designed to detect effect sizes (differences between proportions of indoor-biting and outdoor-biting vectors) as low as 20%, with at least 80% power and 95% confidence limits. As a secondary outcome measure, all *Anopheles* mosquitoes caught were assessed using enzyme-linked immunosorbent assays (ELISA), to estimate proportions carrying infective *Plasmodium falciparum* in their salivary glands [18, 19]. In order to assess preferences of the malaria vectors to biting humans rather than other vertebrates, and to assess any typical resting behaviours of residual transmission vectors, additional mosquito collections in multiple sites including: human dwellings, outdoor kitchen enclosures, cowsheds, lawns, latrines, vegetation, and containers found nearby houses. Two artificial resting sites (pit traps) were constructed per site in all sites except Charawe. It was impossible to construct pit traps in Charawe because most of its surface area is covered by stones and grit. In addition to the pit traps, resting buckets were used to collect resting mosquitoes in each household. Furthermore, to assess how residual vector populations respond to and are affected by common indoor insecticidal interventions, malaria vectors present in the different study sites were collected at larval stage and brought to the insectary where first filial generation (F1) was reared to adult level. Then, 100 unfed mosquitoes per batch of 25 females *Anopheles*, aged 3–5 days old were tested against 10 different types of insecticides (i.e., deltamethrin, permethrin, alpha cypermethrin, lambda cyhalothrin, bendiocarb, pirimiphos-methyl, malathion, fenitrothion, dieldrin, and DDT) currently recommended for vector control [20].

### Mosquito collection

**Fig. 2:**
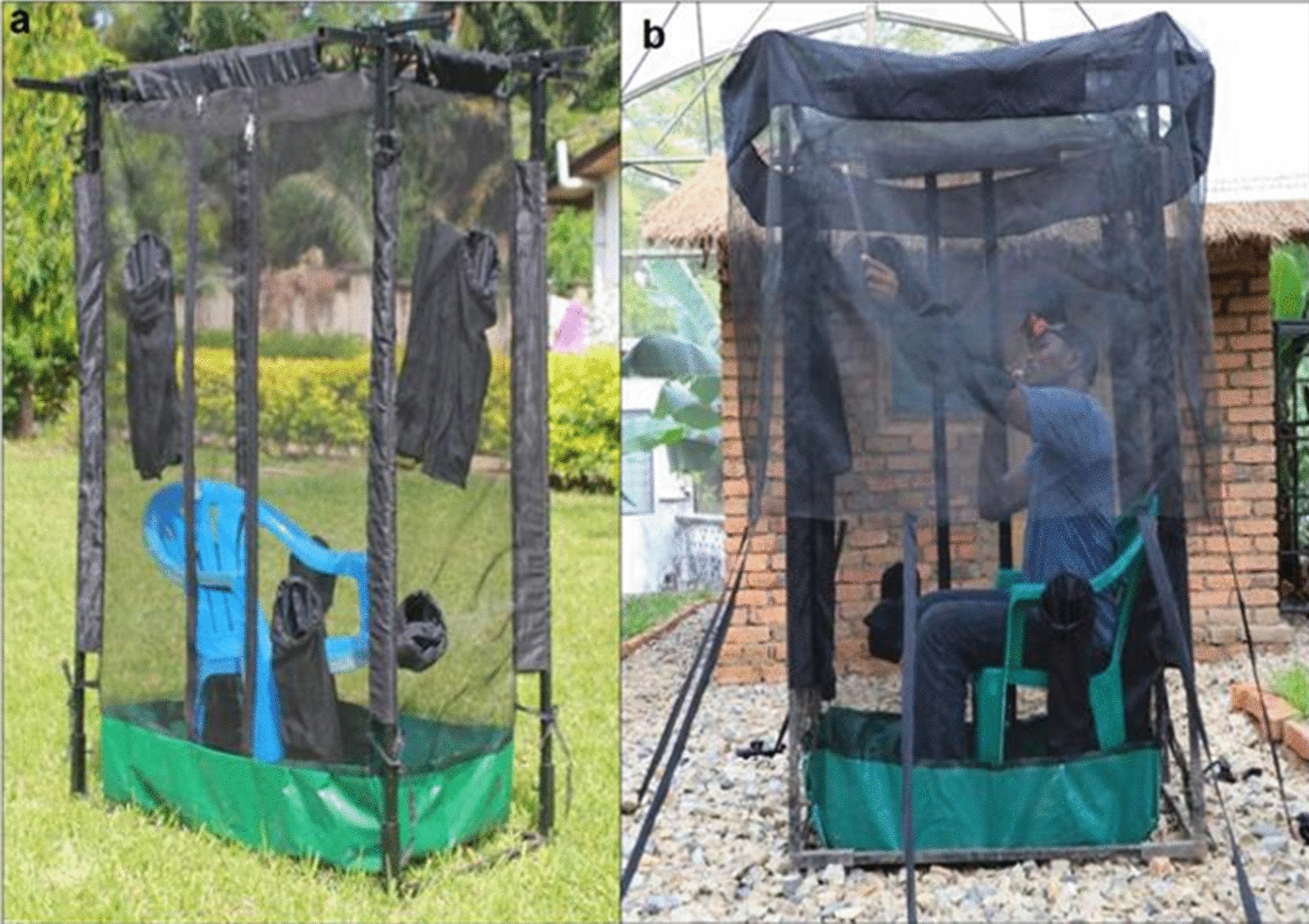
Miniaturized Double Net Trap (DN-Mini). **A** Provides a clear picture of DN-Mini trap and **B** show a volunteer collecting mosquito while seating down but not freely exposed to mosquitoes

Mosquitoes were collected inside and outside households using the miniaturized double net trap (DN-Mini) for 13 h (6:00 pm–7:00 am) as shown in Fig. 2. The DN-Mini is an adaptation of the original bed net trap design [21], which was recently redesigned by Tangena et al. in Lao PDR [16]. The miniaturized double net trap (DN-Mini) was designed to improve comparative mosquito sampling indoors and outdoors while protecting the volunteer collecting mosquitoes (Fig. 2). DN-Mini is constructed by using UV-resistant fibreglass netting materials, on a wooden or metal frame and canvas base as illustrated in Fig. 2. Its size is 60 cm width, 100 cm length and 180 cm height. It has an inner and outer chamber. Volunteers sit in the protective inner chamber and attract host-seeking mosquitoes. Host-seeking mosquitoes attempting to reach the volunteer in the inner chamber are temporarily trapped between the layers, from where they can be retrieved periodically. The inner wall has multiple sleeves through which the volunteers can safely retrieve the mosquitoes in the outer compartment using siphons. The DN-Mini provides an exposure free method for conducting human baited mosquito sampling contrary to the standard method for mosquito sampling, the human-landing catches (HLC), which does not guarantee the safety of volunteers. Additional collections were done indoors using prokopack aspirators to retrieve mosquitoes from walls and ceiling surfaces, and outdoors using resting bucket traps and artificially constructed pit traps.

### Mosquito sampling and processing

Paper cups containing hourly collection of mosquitoes were placed inside a refrigerator (2–7 °C) for ≤ 5 min every morning after a night of collection. The killed mosquitoes were then sorted morphologically into different taxa and sex using dichotomous taxonomic keys [22, 23], and female *Anopheles* were visually classified as being unfed, partially fed, fully fed, or gravid.

Subsamples of individual female *Anopheles* mosquitoes from each hourly catch were stored inside a microcentrifuge tube containing a cotton wool and silica gel beneath for further molecular analyses for *An. gambiae *sensu lato (s.l.) species identification [24], sporozoite infection status, and blood meal status. In cases where hourly catches contained more than 30 mosquitoes, the first 30 mosquitoes were stored individually and all the rest were stored in batches of 10 mosquitoes. The field data and laboratory results were recorded electronically using tablets, linked, cleaned, and stored in a secure web-based database application, the Mosquito Database Management System (MosquitoDB) accessed via www.mosquitodb.io formerly known as the Ifakara Entomology Bioinformatics System (IEBS). The system was designed based on a generic schema described specifically for the purpose of classifying and processing data and samples relating to most entomological studies [25].

### Insecticide susceptibility tests

To assess how residual vector populations respond to and are affected by common indoor insecticidal interventions, we carried out insecticide susceptibility assays using WHO standard procedures and kits [20] for adult female *Anopheles* mosquitoes from four study sites with the highest mosquito abundances to test for the presence of resistance to commonly used insecticides. The study sites where *Anopheles* mosquito larvae collection was performed are Mbaleni, Tunduni, Miwani, and Donge-Mchangani. Mosquito insecticide susceptibility tests were not carried out against mosquitoes from Bwejuu and Charawe sites because during larvae sampling no enough number of *Anopheles* larvae were collected from those sites that could be reared into adults first filial generation (F1). Larvae collected from the other four study sites were brought to the laboratory for rearing into adult mosquitoes of (F1). Batches of 25 adult F1 female *Anopheles* (3–5 days old) were tested against 10 different types of insecticides that are currently recommended for vector control using discriminating concentrations of impregnated filter papers as follows: deltamethrin (0.05%), permethrin (0.75%), alpha cypermethrin (0.05%), lambda cyhalothrin (0.05%), bendiocarb (0.1%), pirimiphos-methyl (0.25%), malathion (5%), fenitrothion (1%), dieldrin (4%), and DDT (4%). A total of 100 mosquitoes were exposed per discriminating concentration in four replicates of 25 mosquitoes each and compared to a control with same number of mosquitoes per replicate. In an exposure tube, mosquitoes were held for a total of 1 h in intervals of 10, 15, 20, 30, 40, 50, and 60 min. After the first hour of exposure, mosquitoes were transferred to non-insecticide treated, clean, holding tubes and observed for a further 20 min [20]. After 80 min (initial 60 min + further 20 min) of knockdown monitoring, all mosquitoes were transferred to non-insecticide treated, clean, holding tubes, kept for 24 h, and provided with 10% glucose solution, after which mortality was monitored and recorded. All these procedures were performed in the laboratory under average ambient temperatures of 26 ± 2 °C and a relative humidity of 78 ± 3% in both bioassay rounds.

Percentage knockdown in the observed mosquitoes was recorded immediately for each time interval, and mosquito mortality in each bioassay was expressed as the proportion of dead mosquitoes to total exposed, for each tested insecticide. Execution and interpretation followed WHO test procedures for insecticide resistance monitoring in malaria vector mosquitoes [20].

Furthermore, commonly used LLINs were tested using cone bioassays for their bio-efficacy and residual activity in killing resistant mosquitoes. LLINs were collected from a few individuals from in our study sites. Five separate sections were cut off from the LLINs and placed into a cone with resistant mosquitoes and the numbers of mosquitoes that are knocked down were recorded.

### Statistical analyses

Data on collected mosquitoes pertaining to *An. gambiae* s.l. were analysed using R statistical package version 3.6.1 [26]. The biting rates for mosquitoes caught indoor versus outdoor were calculated per night as an estimate of risk of exposure of individuals to mosquitoes and a predictor of residual malaria transmission. The difference between indoor and outdoor host-seeking mosquitoes’ behaviours were analysed using Generalized Linear Mixed Models (GLMMs) based on the glmer function under the lme4 R package. Mosquitoes were modelled as a response variable following a negative binomial distribution for count data to account for the over-dispersion when sampling wild mosquitoes with location (indoor/outdoor) of collection as a fixed numeric variable. A household number was used as a random variable that treated each paired indoor/outdoor collection as an independent collection unit. The round of collection and household ID were used as random variables to best account for sampling bias. Shehias with zero mosquito collection were not included in the model fitting.

For insecticide susceptibility tests, knock down time and mortality were considered for every discriminating concentration. Mortality was calculated as the percentage of mosquitoes’- dead-’ post 24 h’ exposure to insecticide, and the results were assessed according to WHO testing procedure for insecticide resistance monitoring in malaria vectors [20]. Mortality rates between 98 and 100% indicated full susceptibility, 90–97% was suggestive of resistance requiring further investigation and mortality rates less than 90% confirmed the existence of resistance [20]. The Abbot formula was used to correct mortality rates in the control.

### Ethical considerations

The study approval was granted by the Johns Hopkins Bloomberg School of Public Health Institutional Review Board (IRB No: 00007390), the Ifakara Health Institutional Review Board (IHI/IRB/No: 035-2016), and the Zanzibar Medical Research Ethical Council (ZAMREC/0005/OCT/2016) in October 2016. The study was granted an extension by the Ifakara Health Institutional Review Board (IHI/IRB/EXT/No: 27-2017) and the Zanzibar Medical Research Ethical Council (ZAMREC/0001/DEC/2017) in October and December 2017, respectively.

Benefits and potential adverse risks associated with the study were explained to the household members. The head of household consented for mosquito collection to be carried out in his/her household through signing an informed consent form. Volunteers for mosquito collection also consented through signing an informed consent form. Before participating in mosquito collection, volunteers were tested for malaria per round of collection and those who were found to be infected were treated with Coartem™ (Artemether–Lumefantrine). On top of that, volunteers were provided with chemoprophylaxis Malarone™ (Atovaquone–Proguanil). In general, volunteers were not exposed to free flying mosquitoes since they were using Miniaturized Double Net that ensured protection as described in method section above. Throughout the study, only one volunteer from Miwani site was tested malaria positive during mosquito sampling. He was given treatment and reported to case management unit (ZAMEP) for further surveillance.

## Results

### Mosquito population and species composition

A total of 26, 365 of mosquitoes were caught of which, 94% were Culicine mosquitoes and 6% were Anopheline mosquitoes. Female Anopheles mosquitoes comprised 95.56% *An. gambiae* s.l, 4.32% *Anopheles squamosus*, 0.062% *Anopheles pharoensis* and 0.062% *Anopheles coustani.* PCR analysis on *An. gambiae* complex indicated that 98.60% were *An. arabiensis*, 0.7% *Anopheles merus* and 0.7% were *Anopheles gambiae* s.s. The number of mosquitoes collected varied per site with Mbaleni having the highest number of mosquito while Charawe and Bwejuu had zero anopheline mosquito catch as shown in Table 1.

**Table 1:** Showing the of number of mosquitoes caught per site

Study site	Mosquito species	Indoor methods			Outdoor methods		Total
		DN	PK	CDC	DN	PK	
Bwejuu	An. species	0	0	0	0	0	0
	Culicines	2402	86	1054	2569	59	6170
Charawe	An. species	0	0	0	0	0	0
	Culicines	1701	17	496	1531	73	3818
Tunduni	An. species	0	1	2	0	8	11
	Culicines	245	8	60	294	133	740
Miwani	An. species	24	2	62	90	85	263
	Culicines	955	20	734	1103	260	3072
Donge Mchangani	An. species	9	0	3	21	213	246
	Culicines	2572	12	1320	3110	210	7224
Mbaleni	An. species	62	6	766	137	106	1077
	Culicines	904	12	1721	1005	102	3744
	Overall total	8874	164	6218	9860	1249	26,365

### Biting pattern and resting preferences

Using a standardized free exposure trap (DN-Mini), a large number of Anopheline mosquitoes were collected outdoor compared to indoor (248 versus 95, respectively) as shown in Table 2.

**Table 2:** Outdoor vs indoor Anopheline mosquitoes caught using DN-Mini

An. specie	Indoor	Outdoor	Total
*An. gambiae* s.l.	90	199	289
*An. squamosus*	5	48	53
*An. coustani*	0	1	1
*An. pharoensis*	0	0	0
Overall total	95	248	343

**Fig. 3:**
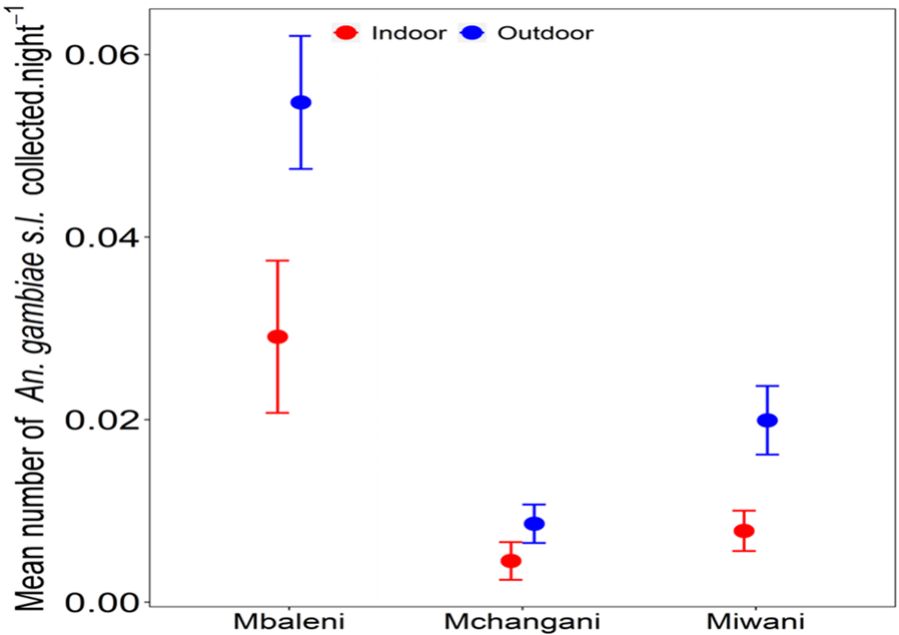
Mean number of *An. gambiae* s.l. collected indoor and outdoor per night in Mbaleni, Donge Mchangani and Miwani

The difference in *An. gambiae* s.l. proportions between indoor and outdoor—mosquito collections were compared using GLLMs assuming a negative binomial distribution. The proportion of outdoor host-seeking mosquitoes was computed as the number of mosquitoes collected from outdoor divided by the overall number of mosquitoes collected indoor and outdoor using DN-Mini. The analysis was only performed on Miwani and Mbaleni. Figure 3 presents the mean number of *An. gambiae* s.l. collected per night indoor and outdoor for the three shehias with reasonable number of mosquitoes caught.

In Miwani, the proportion of mosquitoes caught outdoor is statistically significant and nearly double (RR: 1.942 [1.03, 3.772], 95% CI, p value < 0.05) as compared to indoor (RR: 0.000254 [6.77 * 10^−6^, 0.00244], 95% CI, p value < 0.001). Similarly, in Mbaleni, the proportional of mosquito caught outdoor is statistically significant and double (RR: 2.42 [1.41, 4.25], 95% CI, p value < 0.001) compared to indoor (RR: 0.000123 [1.82^−07^, 0.00198], 95% CI, p value < 0.001).

In addition, 90% of collected mosquitoes were caught during wet season. A similar statistical model used above was used to examine the difference in mosquito bites between dry and wet season by using season as a fixed variable for the same three shehias. In Miwani, the mosquito caught during the dry season (RR: 3.5^−05^ [3.39^−07^, 7.28^−04^], 95% CI, p value < 0.001) is significantly different from the wet season (RR 4.18 [1.32, 4.23^03^], 95% CI, p value < 0.03). In Mbaleni, the mosquito biting rate during the dry season (RR: 0.000124 [1.2^−07^, 5.23^−03^], 95% CI, p value < 0.001) is significantly different from the wet season (RR 4.66 [4.64^−03^, 2.9^03^], 95% CI, p value < 0. 55).

**Fig. 4:**
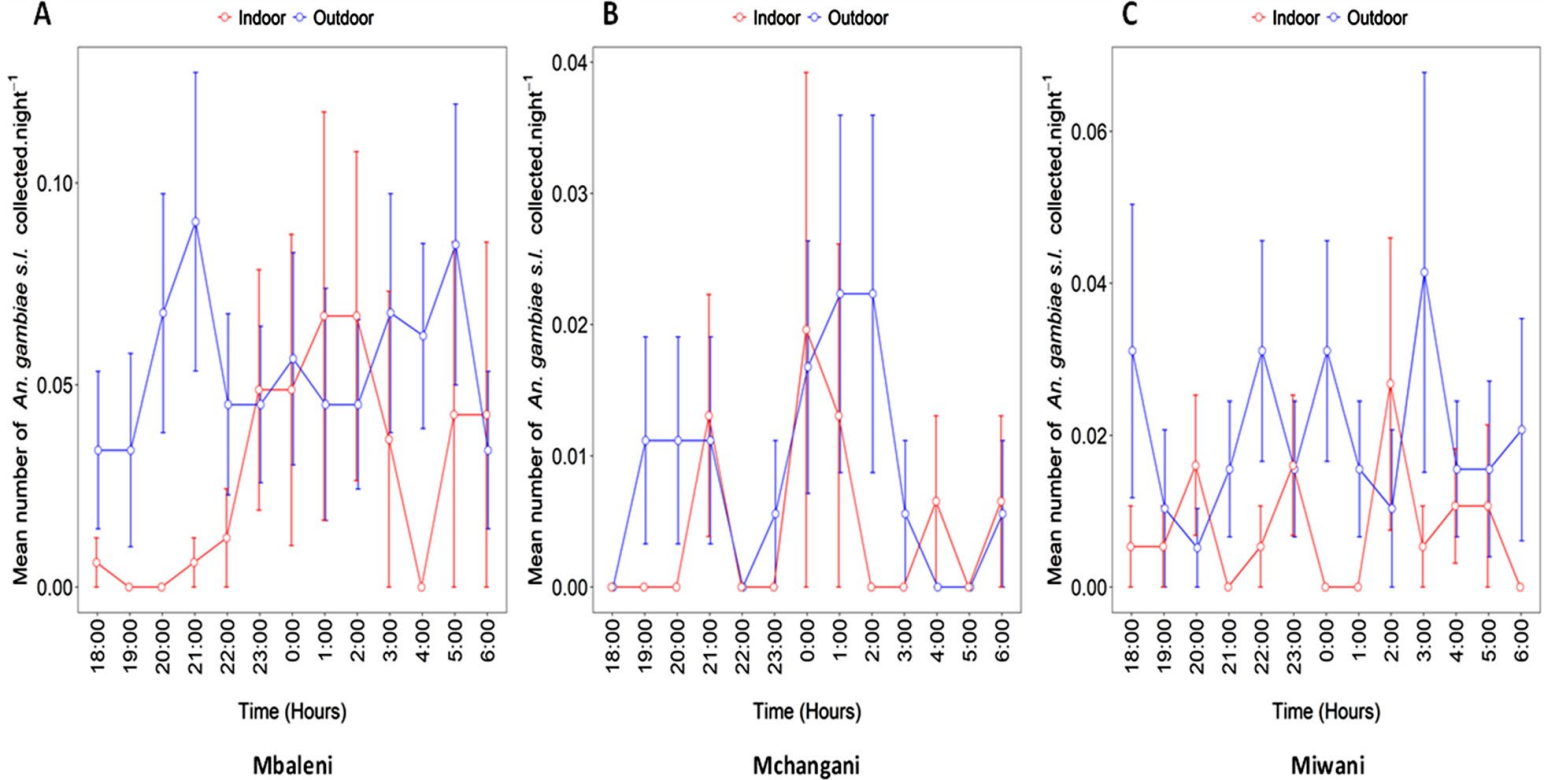
Hourly mean number of *An. gambiae* s.l. caught by DN-Mini in Mbaleni (**A**), Donge (**B**), and Miwani (**C**)

As indicated in Fig. 4, the highest frequency of mosquito bites occurred at 2100 h and 0500 h outdoor but at 0100 h and 0200 h indoor in Mbaleni (panel A), at 0100 h and 0200 h outdoor but at 0000 h indoor in Donge-Mchangani (panel B), at 1800 h and 0300 h outdoor but at 0200 h indoor in Miwani (panel C). While, the lowest frequency of mosquito bites occurred indoors in the early evening and early morning.

Overall for the three sites, the mean numbers of *An. gambiae* s.l. caught by DN-Mini show that the highest frequency of mosquito bites occurred at 1800 h, 2100 h, 0100 h, 0200 h, and 0500 h for outdoor biting mosquitoes and at 0000 h, 0100 h, and 0200 h for indoor biting mosquitoes. While, the lowest frequency of mosquito bites occurred indoors in the early evening and early morning (Fig. 4).

Furthermore, under this study outdoor resting behaviour of mosquitoes was also assessed. The artificially created resting sites (pit-traps) collected 415 Anopheline mosquitoes while the other resting traps (resting buckets) placed indoors did not collect any Anophelines. However, indoor and outdoor resting behaviour could not be directly compared as different trapping methods were used.

### Sporozoite rate and blood meal host preferences

**Fig. 5:**
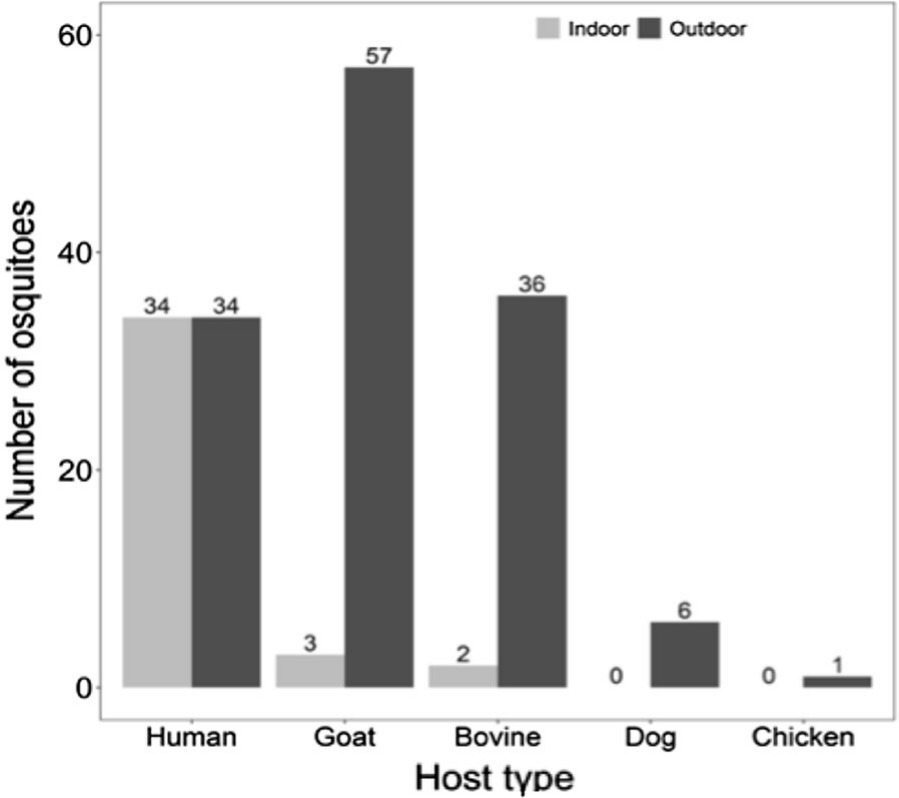
Showing blood meal host preferences displayed by *An. gambiae* s.l. caught by MD-Min both outdoor and indoor dwellings

All mosquitoes subjected to sporozoite ELISA analysis were negative with malaria sporozoite. Using the same technique but of different procedures to quickly analyze the blood meal host preferences it is observed that approximately 60% of blood-fed mosquitoes obtained blood from domestic animals (Goat, Bovine, Dog and Chicken) compared to 40% from human blood (Fig. 5). Table 3 presents the distribution of non-human hosts from the six shehias based on the survey from the human behaviour component of the study.

**Table 3:** Distribution of non-human hosts from the study sites

Study sites	Non-human host					
	Chicken	Duck	Goat	Cow	Cat	Pigeon
Miwani	115	2	4	16	0	6
Mbaleni	241	22	13	43	2	5
Bwejuu	5	7	0	1	0	0
Donge Mchangani	436	23	3	41	0	0
Tunduni	140	6	12	33	0	3
Charawe	68	0	0	12	0	0
Overall total	1005	60	32	146	2	14

*Anopheline* mosquitoes feed on human blood relatively equally indoor and outdoor (Fig. 5). Contrary to equal balance of mosquitoes with human blood, more mosquitoes with blood from goat, bovine, dog and chicken were sampled from outdoor dwellings.

### Mosquito susceptibility and intensity/level of insecticide resistance

Mosquitoes tested against selected insecticides from the pyrethroid class resulted in non-uniform resistances status across the four sites. Under pyrethroid class, mosquitoes were found susceptible to Permethrin (0.05%) at Donge-Mchangani North part of Unguja, Mbaleni and Tunduni in Central part of Unguja. Apart from permethrin (0.05%), the tested mosquitoes were also susceptible to Deltamethrin (0.05%) at Mbaleni Central part of Unguja. Furthermore, mosquitoes at different sites displayed middle outcome lying between susceptible and resistant called possible resistance indication according to WHO insecticide susceptibility procedures update 2016. Mosquitoes that were tested and indicated suggestive resistance were tested against Deltamethrin (0.05%) at Donge-Mchangani North part of Unguja, Lambda-cyhalothrin (0.05%) at both Mbaleni and Tunduni in Central part of Unguja.

Moreover, mosquitoes were tested and confirmed resistant against lambda-cyhalothrin (0.05%) at both Donge-Mchangani and Miwani, alpha-cypermethrin (0.05%) at Donge-Mchangani, Mbaleni, Miwani, and Tunduni, and permethrin (0.75%) at Miwani as shown in Tables 4, 5, 6, and 7.

**Table 4:** Susceptibility status at Mbaleni site

Insecticide class	Insecticide name	Knockdown (%) 60 min	Mortality (%) 24 h	Susceptibility status
Pyrethroid	Deltamethrin (0.05%)	100	100	Susceptible
	Permethrin (0.75%)	100	100	Susceptible
	Lambda-cyhalothrin (0.05%)	100	97	Possible resistance
	Alpha-cypermethrin (0.05%)	88	60	Confirmed resistance
Carbamates	Bendiocarb (0.1%)	100	100	Susceptible
Organophosphates	Pirimiphos-methyl (0.25%)	96	100	Susceptible
	Malathion (5%)	100	100	Susceptible
	Fenitrithion (1%)	80	100	Susceptible
Organochlorine	Dieldrin (4%)	99	100	Susceptible
	DDT (4%)	100	100	Susceptible

**Table 5:** Susceptibility status at Miwani site

Insecticide class	Insecticide name	Knockdown (%) 60 min	Mortality (%) 24 h	Susceptibility status
Pyrethroid	Deltamethrin (0.05%)	99	73	Confirmed resistance
	Permethrin (0.75%)	14	21	Confirmed resistance
	Lambda-cyhalothrin (0.05%)	93	82	Confirmed resistance
	Alpha-cypermethrin (0.05%)	95	97	Possible resistance
Carbamates	Bendiocarb (0.1%)	100	100	Susceptible
Organophosphates	Pirimiphos-methyl (0.25%)	84	100	Susceptible
	Malathion (5%)	100	100	Susceptible
	Fenitrithion (1%)	68	100	Susceptible
Organochlorine	Dieldrin (4%)	92	100	Susceptible
	DDT (4%)	100	100	Susceptible

**Table 6:** Susceptibility status at Tunduni site

Insecticide class	Insecticide name	Knockdown (%) 60 min	Mortality (%) 24 h	Susceptibility status
Pyrethroid	Deltamethrin (0.05%)	76	26	Confirmed resistance
	Permethrin (0.75%)	95	100	Susceptible
	Lambda-cyhalothrin (0.05%)	97	96	Possible resistance
	Alpha-cypermethrin (0.05%)	79	96	Susceptible
Carbamates	Bendiocarb (0.1%)	99	100	Susceptible
Organophosphates	Pirimiphos-methyl (0.25%)	93	100	Susceptible
	Malathion (5%)	100	100	Susceptible
	Fenitrithion (1%)	58	100	Susceptible
Organochlorine	Dieldrin (4%)	94	100	Susceptible
	DDT (4%)	100	100	Susceptible

**Table 7:** Susceptibility status at Donge Mchangani site

Insecticide class	Insecticide name	Knockdown (%) 60 min	Mortality (%) 24 h	Susceptibility status
Pyrethroid	Deltamethrin (0.05%)	97	97	Possible resistance
	Permethrin (0.75%)	100	100	Susceptible
	Lambda-cyhalothrin (0.05%)	83	85	Confirmed resistance
	Alpha-cypermethrin (0.05%)	94	81	Confirmed resistance
Carbamates	Bendiocarb (0.1%)	100	100	Susceptible
Organophosphates	Pirimiphos-methyl (0.25%)	89	100	Susceptible
	Malathion (5%)	100	100	Susceptible
	Fenitrithion (1%)	96	100	Susceptible
Organochlorine	Dieldrin (4%)	100	100	Susceptible
	DDT (4%)	100	100	Susceptible

Contrary to outcome variation observed under Pyrethroid class insecticides, representative insecticides from the other three classes of insecticides (i.e. organochlorine, organophosphate and carbamate) were also tested and resulted in uniform outcomes where all the tested mosquitoes were susceptible against the insecticides to which they were exposed (Tables 4, 5, 6, and 7).

Similar to susceptibility test outcomes, the intensity of resistance test results was non-uniform. The intensity of resistance varied across the four sites and the insecticide tested as shown in Table 8.

**Table 8:** Variation of intensity of resistance across the four study sites

Study site	Insecticide name	Intensity of resistance
Donge	Deltamethrin	Low intensity
	Alpha-cypermethrin	Low intensity
Mbaleni	Lambda-cyhalothrin	Moderate to high intensity
	Alpha-cypermethrin	Moderate to high intensity
Miwani	Alpha-cypermethrin	Low intensity
	Lambda-cyhalothrin	Low intensity
	Permethrin	Low intensity
	Deltamethrin	Moderate to high intensity
Tunduni	Alpha-cypermethrin	Low intensity
	Lambda-cyhalothrin	Low intensity
	Deltamethrin	Low intensity

Generally, the intensity of resistance across the four sites ranged from low intensity and, moderate to high intensity e.g. mosquitoes tested against Deltamethrin (0.25%) at Miwani, Tunduni, and Donge-Mchangani had different outcome between the mentioned sites. Mosquitoes tested at Miwani resulted in moderate to high-intensity resistance while mosquitoes exposed to deltamethrin (0.25%) at Donge-Mchangani and Tunduni displayed low-intensity resistance. This kind of variation is seen also against other insecticides and at different sites as presented in Table 8.

## Discussion

These findings highlight potential entomological drivers contributing to residual malaria transmission in Zanzibar and the testing of complementary novel techniques that can be used to target outdoor biting and resting mosquitoes. Key findings include: a high proportion of outdoor biting and resting behaviour, high levels of zoophilic behaviour, and varying levels of pyrethroid resistance across sites. All these factors could contribute to ongoing local malaria transmission in Zanzibar.

After eight rounds of indoor and outdoor mosquito collection in 135 households across six Shehias in four districts only a total of 704 malaria vectors were collected. In most trap nights, the average number of malaria vectors caught was zero especially in dry season. This indicates that the mosquito density is very low in Zanzibar especially in the dry season. ZAMEP has also reported the very low numbers of malaria vectors collection from 2012 to 2018 (ZAMEP reports, unpublished data). Malaria vector reduction is likely to be attributed to the continuous use of LLINs as well as implementation of IRS in areas with high density of malaria vectors [27]. In addition, a majority of malaria vectors were *An. arabiensis* (98.4%), mostly caught outdoors (85%), which is an opportunistic species [28–30] and it is not surprising that out of 173 blood-fed mosquitoes-39.30% obtained blood from human and 60.70% from non-human hosts (i.e., 34.68%, 21.97%, 3.47%, and 0.58% from Goat, Bovine, Dog and Chicken respectively). In general, a large significant number of mosquitoes with blood from non-human hosts were sampled from outdoor dwellings. In contrast, mosquitoes fed equally outdoor (50%) and indoor (50%) on human blood—exhibiting *An. arabiensis* feeding behaviour [31, 32]. Similar findings on outdoor and indoor biting preference and malaria vector species composition were also obtained by ZAMEP as presented in their entomological report from 2012 to 2018—indicating high number of *An. arabiensis* compared to *An. gambiae* s.s. [33]. This significant reduction of *An. gambiae* s.s. in the island might be due to wide use of indoor interventions (i.e., (LLINs) and (IRS)) [27]. *Anopheles arabiensis* is becoming a major malaria vector in Zanzibar and its increasing role in malaria transmission has also been reported in various studies in Tanzania [34] and in Kenya [35]. Sporozoite ELISA analysis indicates that all mosquitoes tested were negative for the malaria parasite. Nevertheless, significant outdoor biting behaviour can potentially indicate the high risk of outdoor malaria transmission in Zanzibar. In addition, it was also observed that the artificially created outdoor resting sites collected a large number of *Anopheline* mosquitoes as compared to the number collected resting indoors- and this has also been demonstrated in ZAMEP’s entomological reports (2013–2017). This might also highlight the mosquito behaviour change from resting in indoor dwellings e.g. ceiling, walls to outdoor dwelling like the dug hole and other areas with favourable resting conditions e.g. darkness. The implementation of indoor core interventions i.e. LLINs and/or IRS as previously demonstrated in some studies as one of the driving forces of mosquitoes behavioural change [31, 36, 37].

In understanding that insecticide resistance may impact the effectiveness LLINs and IRS which are widely used in Zanzibar—insecticide resistance tests based on WHO procedures were carried out [20]. The tests were carried on commonly used insecticide based on samples from only four sites with the highest mosquito densities. The findings indicate that testing outcomes for pyrethroid class insecticides across the four sites were not uniform. The susceptibility tests indicated that mosquitoes were susceptible to some insecticides, displayed possible resistance to others depending on the insecticide tested. Insecticide intensity outcomes also varied across the study sites. Generally, the intensity of resistance across the four sites ranged from low intensity, moderate to high intensity. The susceptibility and intensity variation are also reported in ZAMEP reports for Unguja site but it is uniform in Pemba study sites where mosquitoes have displayed high level of resistance to pyrethroid class insecticide (ZAMEP report 2018). These variations might be explained by several facts including tendency of net use in the particular site, level of urbanization as well as agricultural activities. The use of different insecticides in agricultural activities might trigger high selection pressure of insecticide resistance in one site compared to another site [38, 39].

Insecticide resistance variation (heterogeneity) across the four study sites highlight the need for Zanzibar Malaria Elimination Program (ZAMEP) to explore the focalised insecticide use. Exploration of insecticide resistance variation site by site might be an important strategy of managing insecticide resistance variation in different sites. In contrast, mosquitoes were susceptible to all representative insecticides from the other three classes (i.e., organochlorine, organophosphate and carbamate) across all the four sites. The insecticide resistance carried out by ZAMEP in 2018 in three different sites in Unguja are consistent with our findings (ZAMEP report 2018, unpublished data). Insecticide resistance in African malaria vectors have also been reported in other malaria endemic settings [40–44]. The resistance levels and mechanisms to African malaria vectors have been documented all over in malaria endemic settings [44–47]. The wide spread of insecticide resistance affects both malaria control and transmission [48–51]. This on-going mosquito resistance to pyrethroids highlights the need to consider other options for the insecticide used for LLINs and IRS programs.

Recently, the addition of a synergist, piperonyl butoxide (PBO), to pyrethroid nets has been investigated. Available evidence suggest that pyrethroid-PBO nets are more effective than standard pyrethroid nets in settings with insecticide resistance [52, 53]. While pyrethroid-PBO nets can help to maintain protection in some settings, they should not be considered a tool for insecticide resistance management, rather broader insecticide resistance management strategies are essential.

Most entomological studies will normally calculate and report entomological inoculation rate (EIR) which is the practical indicator of human exposure mosquitoes bites infected with transmissible sporozoite-stage malaria parasites [54]. This indicator can be used to assess the impact of vector control tools (VCTs) and it is important for VCTs to sustainably reduce EIRs to levels below 1 in order to interrupt malaria transmission [55]. In this study, EIR was not calculated due to the absence of any sporozoite positive mosquito. The findings from ZAMEP’s mosquito routine collections in Zanzibar indicated that five sporozoite positive *Anopheles arabiensis* mosquitoes detected by CSP ELISA have been confirmed in the last 6 years (2012–2018) in Unguja. However, no positive sporozoite mosquitoes have been detected in Pemba in the same period, (ZAMEP report, unpublished data). This indicates that the transmission is declining to the extent that it is becoming difficult to estimate EIR or it might require using advanced techniques for sporozoites detection in low transmission settings.

A key limitation of the study was the inability to detect sporozoite rates from blood-fed mosquitoes collected. CSP ELISA was used for sporozoite detection and the study found no sporozoite positive mosquitoes. As a result, it was not possible to calculate the current EIR value (given sporozoite infection rate as a factor). This could reflect the characteristically low transmission of malaria in Zanzibar along with *Anopheles* mosquito density. This limitation highlights the need of applying more advanced sporozoite detection methods (i.e., PCR ELISA) that could allow the detection of sporozoite rates. Higher resolution sampling in hot spot areas could be helpful in catching sporozoite positive mosquitoes. These findings illustrate that, in areas of persistent low malaria transmission more powerful methods may be needed to detect sporozoite infection rates. In similar settings, entomological studies alone may be inadequate for making a conclusion regarding the presence of local malaria transmission but will require using other techniques such as detection of malaria parasite movement through genetic sequencing.

Another limitation of the study methodology is associated with the type of trap used to collect mosquitoes. Hourly mosquito collections were carried out in the peri-domestic setting using the double net trap (MD-Mini) [16] instead of human landing catch (HLC) which is often considered the gold standard [56]. Therefore, the recorded biting rates may have been impacted by the trapping method used. However, a previous study showed no significant difference between number of *Anopheles* caught by HLC and DN-Mini [16]. The double net trap was used to increase the safety of volunteers during the collection by ensuring that they are not freely exposed to mosquito bites.

Despite these limitations, this study has contributed to understanding the magnitude and entomological drivers contributing to the residual malaria transmission in Zanzibar and areas with similar transmission settings. The study suggests increased outdoor-biting proportions, shifts in peak biting times to early-evening hours, and the rise of pyrethroid-resistant *Anopheles* vectors. Therefore, the findings from this study highlight the need to consider using bednets treated with other insecticide (e.g., a LLIN that incorporates a synergist piperonyl butoxide (PBO) [52], different insecticide for IRS programs in Zanzibar [52] and/or rotation of insecticide application mode [44], and proper implementation of larviciding which target mosquitoes at aquatic stages [57–59]. In addition, complementary tools enhancing indoor protection and especially targeting outdoor transmission have the potential to contribute to eliminating residual malaria transmission in Zanzibar.

## Conclusion

This study suggests that in contexts such as Zanzibar—testing of novel techniques to complement indoor protection and especially targeting outdoor biting and/or resting mosquitoes as well as immature mosquitoes, may be warranted in these settings. If found to be effective, these suggested approaches would complement existing interventions that target mosquitoes biting and/or resting indoors. The study also highlights the need to consider insecticide resistance in selection of core vector control interventions.

## Data Availability

The dataset supporting the conclusions of this article is available upon request in MosquitoDB application accessible via https://www.mosquitodb.io.

## Abbreviations

LLINS: Long lasting insecticidal nets
IRS: Indoor residual spraying
DN: Double net
PCR: Polymerase chain reaction
ELISA: Enzyme-linked immunosorbent assay
WHO: World Health Organization
PBO: Piperonyl butoxide
RDTs: Rapid diagnostic tests
ACTs: Artemisinin-based combination therapy
ZAMEP: Zanzibar Malaria Elimination Program
HLC: Human-landing catch
IEBS: Ifakara Entomology Bioinformatics System
DDT: Dichlorodiphenyltrichloroethane
IRB: Institutional Review Board
ZAMREC: Zanzibar Medical Research Ethical Council
EIR: Entomological inoculation rate
VCTs: Vector control tools
F1: First filial generation
NMCP: National Malaria Control Programme
